# Usefulness of the Waist Circumference-to-Height Ratio in Screening for Obesity and Metabolic Syndrome among Korean Children and Adolescents: Korea National Health and Nutrition Examination Survey, 2010–2014

**DOI:** 10.3390/nu9030256

**Published:** 2017-03-10

**Authors:** Dong-Hyun Choi, Yang-Im Hur, Jae-Heon Kang, Kyoungwoo Kim, Young Gyu Cho, Soo-Min Hong, Eun Byul Cho

**Affiliations:** 1 Department of Family Medicine, Seoul Paik Hospital, Inje University College of Medicine, Seoul 100032, Korea; ksz666@naver.com (D.-H.C.); fmleader@nuri.net (J.-H.K.); kwkimfm@gmail.com (K.K.); jacobel@hanmail.net (Y.G.C.); starsilver1@naver.com (E.B.C.); 2 Department of Internal Medicine, Seoul Paik Hospital, Inje University College of Medicine, Seoul 100032, Korea; raisondetre@hanmail.net

**Keywords:** waist-to-height ratio, children, adolescents, overweight, obesity, metabolic syndrome, cutoff values, body mass index, waist circumference

## Abstract

The aims of this study were to assess the diagnostic value of the weight-to-height ratio (WHtR) for the detection of obesity and metabolic syndrome (MS) in Korean children and adolescents, and to determine the advantages of WHtR as a population-based screening tool in comparison with other obesity indicators, such as body mass index (BMI) and waist circumference (WC). We performed a cross-sectional analysis of data from 3057 children and adolescents (1625 boys, 1332 girls) aged 10–19 years who were included in the fifth Korean National Health and Nutrition Examination Survey (KNHANES, 2010–2012) up to the second year of the sixth KNHANES (2013–2014). Receiver operation characteristic (ROC) curves were generated to determine the optimal cutoff value and accuracy of WHtR for predicting individual obesity indicators or more than two non-WC components of MS. The area under the ROC curve (AUC) is a measure of the diagnostic power of a test. A perfect test will have an AUC of 1.0, and an AUC equal to 0.5 means that the test performs no better than chance. The optimal WHtR cutoff for the evaluation of general obesity and central obesity was 0.50 in boys and 0.47–0.48 in girls, and the AUC was 0.9. Regarding the assessment of each MS risk factor, the optimal WHtR cutoff was 0.43–0.50 in boys and 0.43–0.49 in girls, and these cutoffs were statistically significant only for the detection of high triglyceride and low High-density lipoprotein (HDL) cholesterol levels. When a pairwise comparison of the AUCs was conducted between WHtR and BMI/WC percentiles to quantify the differences in power for MS screening, the WHtR AUC values (boys, 0.691; girls, 0.684) were higher than those of other indices; however, these differences were not statistically significant (boys, *p* = 0.467; girls, *p* = 0.51). The WHtR cutoff value was 0.44 (sensitivity, 67.7%; specificity, 64.6%) for boys and 0.43 (sensitivity, 66.4%; specificity, 66.9%) for girls. There was no significant difference between the diagnostic power of WHtR and that of BMI/WC when screening for MS. Although the use of WHtR was not superior, WHtR is still useful as a screening tool for metabolic problems related to obesity because of its convenience.

## 1. Introduction

Childhood obesity and metabolic syndrome (MS) associated with obesity are considered major health problems worldwide [1]. The global prevalence of obesity has been increasing [2], and the prevalence of obesity in children and adolescents in Korea has also increased from 5.8% in 1997 to 9.7% in 2005, 10.95% in 2007, 10.8% in 2010, and 11.5% in 2014 [3]. Childhood obesity is more than 50% likely to lead to adult obesity [4,5], and it increases the risk of MS, a cluster of cardiovascular risk factors such as visceral obesity and lipid metabolism abnormalities [6]. In addition, since atherosclerosis can begin at an early age [7], obesity and MS in pediatric individuals can continue into adulthood [7,8]; thus, early detection and management of MS is very important for the prevention of cardiovascular disease in adulthood [4,5,9]. 

Detecting MS early requires a long timeframe and is costly, as each parameter of MS must be investigated [10]. In addition, although the prevalence of MS is increasing in pediatric individuals, its prevalence is still low. Therefore, it is not cost effective to conduct blood tests for early detection of metabolic abnormalities in all individuals at risk [11]. For this reason, anthropometric parameters, including body mass index (BMI), waist circumference (WC), and waist-to-height ratio (WHtR), have been suggested as screening tools for use in children and adolescents with cardiovascular risk factors [11]. 

A good screening test should be not only highly predictive but also easy to perform and interpret [12]. BMI and WC have been the most commonly used predictors of obesity and cardiovascular disease to date. Unlike adults, for whom a single obesity criterion is used, both BMI and WC percentiles by race, sex, and age are used to determine obesity in children and adolescents. Therefore, determining the obesity status for medical treatment is difficult using these methods [13,14] and it is not easy for the general population to understand these criteria. On the other hand, WHtR can be applied to a single reference point regardless of race, sex, or age. Consequently, it has been proposed as a population-based screening tool for cardiometabolic risk prediction in large-scale epidemiological studies and during medical examinations [12,15,16].

Several previous studies have shown that WHtR has a high sex match and is a useful index for evaluating abdominal, especially visceral, fat associated with cardiovascular risk factors [17]. In a recent meta-analysis investigating children and adolescents by Lo et al. [12], WHtR was shown to be a good predictor of cardiometabolic risk, and in some studies, its screening capacity was better than that of BMI and WC. However, the overall analysis concluded that the screening ability of WHtR was not significantly better than that of the other two indices. However, it has been suggested that using WHtR rather than BMI and WC may result in easier and more rapid identification of children and adolescents with cardiovascular risk factors [12]. In addition, the measurement and interpretation of WHtR are more convenient than those of the other two indicators [12]. Only one study has confirmed WHtR cutoff values for the assessment of cardiometabolic risk in Korean pediatric patients, performed in 314 obese adolescents visiting an obesity clinic [18]. Regardless of age and sex, a WHtR cutoff value of 0.5 is a significant indicator for predicting cardiovascular metabolic risk [16]. However, some studies have suggested other cutoff values with better sensitivity and specificity as indicators of obesity or metabolic abnormalities in children and adolescents [19,20,21,22,23,24]. Therefore, it is necessary to confirm an appropriate WHtR cutoff value in screening for cardiovascular metabolic disease risk in Korean children and adolescents [20].

In this study, the optimal WHtR cutoff value for screening for obesity and MS among children and adolescents was determined using recent data representative of Korean children and adolescents. Moreover, the usefulness of WHtR as a population-based screening tool for cardiovascular risk factors was compared with that of other obesity indicators including BMI and WC.

## 2. Materials and Methods 

### 2.1. Study Population

In this study, data were analyzed from the fifth (2010–2012) and sixth (2013–2014) Korean National Health and Nutrition Examination Surveys (KNHANES) investigating children and adolescents aged 10–19 years conducted by the Korean Centers for Disease Control and Prevention (KCDC) [3,25,26]. The KNHANES is a nationwide health and nutrition survey performed annually to identify the health behaviors of the Korean population, the current status of chronic diseases, and data concerning food and nutrition consumption [26]. After selecting 20 households by applying a two-stage stratified sampling method using sampling units and households as first and second sampling units, respectively, health surveys, examinations, and nutrition surveys were conducted for household members aged 1 year or older. Of the 4885 children aged 10 to under 19, those who missed physical or blood pressure (BP) measurements or blood test results and those who did not fast for more than 8 h were excluded. Thus, the final study subjects numbered 3057 (1625 boys, 1432 girls). All participants provided informed consent before data collection, and the survey was approved by the KCDC Bioethics Committee (approval numbers: 2010-02CON-21-C, 2011-02CON-06-C, 2012-01EXP-01-2C, 2013-07CON-03-4C, and 2013-12EXP-03-5C in 2010, 2011 2012, 2013, and 2014, respectively). The study protocol was exempt from examination by the clinical examination committee of Seoul Paik Hospital of Inje University (Institutional Review Board no. IIT-2016-318). 

### 2.2. Anthropometric Measurements

#### 2.2.1. Anthropometric Measurements 

All anthropometric measurements were performed by trained individuals using the same methods. The methods performed by the KNHANES are as follows. Height was measured with the subject on his/her side by contacting the subject’s heels, hips, back, and back of the head with the vertical plate of the extensometer (SECA 225, SECA, Hamburg, Germany) while the subject maintained a horizontal gaze. The measuring plate was pressed to the vertex of the head with only enough pressure to press the hair down, and the measurement was recorded to the nearest 0.1 cm. Weight was measured to the nearest 0.1 kg while the subjects wore a disposable gown. Then, BMI was calculated according to the height and weight measurements as follows: BMI = body weight (kg)/height (m^2^). 

Using a tape measure (SECA 200, SECA), WC was measured to the nearest 0.1 cm while the subjects were breathing out. A mark was made using a water-based ink pen at the midpoint between the lower end of the last rib and the upper rim of the iliac crest at the central axillary line. If the degree of obesity was severe, the subject was asked to touch two points (the lower end of the last rib and the upper rim of the iliac crest) directly, or the subject was instructed to lean the upper body forward, and the lower part of the last rib was touched directly. If the two points could not be discerned, WC was measured at a point slightly above the navel. WHtR was obtained by dividing WC by height [27]. 

#### 2.2.2. Blood Pressure 

BP was measured using a mercury sphygmomanometer (Baumanometer Desk Model 0320; WA Baum, Co., Copiague, NY, USA). The subjects were asked to stop smoking at least 30 min prior to the BP measurement and to rest in a sitting position for 5 min before the test. The subjects sat in a chair with a backrest and armrests during the measurements. After palpating the brachial artery of the right arm such that the middle part of the cuff bladder was on top, the cuff was rapidly expanded to the maximum inflation pressure. The valves were regulated such that the pressure dropped constantly at a rate of 2 mmHg/s, and the systolic and diastolic BPs were monitored. The systolic BP was defined as the first Korotkoff sound and the diastolic BP as the fifth Korotkoff sound. BP was measured three times, and the mean of the second and third measurements was used for analysis. 

#### 2.2.3. Laboratory Tests

Blood tests were performed after the subjects had fasted for more than 8 h. Fasting plasma glucose (FPG), total cholesterol, triglyceride, and low-density lipoprotein (LDL)-cholesterol levels were measured using an automatic analyzer (Hitachi Automatic Analyzer 7600, Hitachi, Japan). High-density lipoprotein (HDL) cholesterol was defined as the value calculated from the conversion formula for standardization [26]. 

### 2.3. Diagnostic Criteria

Obesity was defined using the 2007 Pediatric Adolescent Standard Growth Chart [28] from the Center for Disease Control and the Association of Korean Pediatrics. Weight classifications according to BMI diagnostic criteria were: normal weight, ≥the 5th percentile and <the 85th percentile by sex and age; overweight, ≥the 85th percentile and <the 95th percentile; and obesity, ≥the 95th percentile or BMI ≥25 kg/m^2^ [29]. Moreover, obesity was defined as WC ≥the 90th percentile by sex and age [30,31]. The risk factors for MS were defined according to the modified National Cholesterol Education Program, Adult Treatment Panel III (NCEP ATP III) criteria [30]: (1) abdominal obesity, WC ≥ the 90th percentile by sex and age; (2) high triglyceride, fasting triglyceride level ≥110 mg/dL; (3) low HDL-cholesterol: HDL level <40 mg/dL (fasting); (4) high BP, systolic BP ≥130 mmHg or diastolic BP ≥85 mmHg for subjects >18 years of age, and systolic or diastolic BP ≥the 90th percentile by age, sex, and height for subjects <18 years of age; and (5) high FPG, FPG ≥110 mg/dL. MS was diagnosed when more than three of the above risk factors were detected. Non-WC components of MS was defined as having all of the above risk factors for MS except abdominal obesity. 

### 2.4. Statistical Analysis

All analyses were performed for all participants and separately for boys and girls using the composite sample design and weighting of the KNHANES, except for receiver operating characteristic (ROC) curve analysis. The independent *t*-test and chi-square test were used to analyze differences according to sex. Continuous variables were expressed as means and standard errors and nominal variables as frequencies and percentages (standard error). ROC analysis was used to determine the optimal threshold and accuracy of WHtR in predicting the obesity indices (BMI and WC percentiles) and individual or more than two non-WC components of MS, including high FPG, high BP, high triglyceride, and low HDL cholesterol. The optimal threshold was determined using the Youden index (maximum value of (sensitivity + specificity − 1)), and all sensitivities, specificities, and J-values (sensitivity + specificity − 1) are presented [32]. The sensitivity, specificity, positive predictive value, negative predictive value, and J-values of 0.5 and 0.6 that were suggested as the optimal WHtR cutoff levels for predicting cardiovascular disease in previous studies [16,33], and this study’s optimal cut-off values for predicting two or more non-WC components of MS are presented. To determine differences in the predictive values of abdominal obesity indices (WHtR, WC, and BMI) for more than one, more than two, or individual MS risk factors. WC and BMI were stratified to calculate percentiles, and ROC curve analysis was performed subsequently. The area under the curve (AUC) values were compared using non-parametric methods to confirm significance [34]. An AUC ≥0.5 is less accurate but remains useful for screening tests, an AUC >0.7 indicates an accurate value for screening, and an AUC >0.9 indicates a very accurate value [35,36]. Moreover, ROC analysis can be applied to measure differences in AUC values, thereby enabling identification of the most powerful variables [37]. ROC analysis was employed to avert assumptions of normal distribution and to analyze multiple predictors concurrently [37]. Continuous variables were adjusted into the binary classification (0 = normal, 1 = abnormal) according to the given threshold value [37]. In addition, calculations were corrected for multiple testing by computing 95% confidence intervals (CIs) [37]. Statistical analysis of the AUC difference between the three indexes (BMI percentile, WC percentile, WHtR) was performed using the STATA software ver. 12.0 (StataCorp LP, College Station, TX, USA). Other statistical analyses were performed using the SAS software ver. 9.4 (SAS Institute Inc., Cary, NC, USA). *p* < 0.05 was considered to indicate statistical significance. 

## 3. Results

The general characteristics of the study subjects are shown in Table 1, according to sex. The mean age of the subjects was 14.26 ± 0.0 years, and the mean BMI was 20.96 ± 0.12 kg/m^2^ for boys and 20.42 ± 0.11 kg/m^2^ for girls. The WC and WHtR values were significantly larger in boys than girls (girls, 67.29 ± 0.2 cm and 0.427 ± 0.00, respectively; boys, 71.33 ± 0.33 cm and 0.432 ± 0.00, respectively). Overall, 12.0% of the subjects (14.1% of boys and 9.7% of girls) were obese, and 6.0% (4.4% of boys and 7.9% of girls) were overweight. The prevalence of obesity was higher in boys, and the prevalence of overweight was higher in girls. There were no significant differences in the prevalence of underweight or normal weight participants according to sex. Overall, a high triglyceride level was the most common risk factor, while a high fasting blood glucose level was the least common risk factor among the study subjects. Apart from central obesity, all other MS components were more prevalent among boys than girls, albeit without statistical significance; however, low HDL-cholesterol levels were significantly higher in boys than girls. In this sample, 45.6% of the subjects (boys, 48.4%; girls, 42.5%) had one non-WC components of MS, and 12.4% (boys, 14.4%; girls, 10.2%) had two non-WC components of MS, while 6.2% (boys, 7.0%; girls, 5.2%) were diagnosed with MS. There were no significant differences in MS prevalence between boys and girls. 

Table 2 shows the results of the ROC curve analysis performed to identify the optimal WHtR cutoff values for predicting general obesity, central obesity, and MS. The optimal WHtR cutoff value for predicting obesity according to BMI was 0.47 for both sexes. The AUC for predicting obesity according to BMI in boys and girls was 0.950 (sensitivity, 91.1%; specificity, 85.9%) and 0.965 (sensitivity and specificity, 90.2%), respectively.

The optimal WHtR cutoff value for predicting obesity according to WC >the 90th percentile was 0.50 for boys, with an AUC of 0.990 (sensitivity, 97.5%; specificity, 94.4%), and 0.48 for girls, with a AUC of 0.985 (sensitivity, 94.6%; specificity, 94.6%) (Table 2). The optimal WHtR cut-off values for predicting each MS risk factor ranged from 0.43 to 0.50 in boys and 0.43 to 0.49 in girls; these values were statistically significant only for hypertriglyceridemia and low HDL cholesterolemia (Table 2). The optimal WHtR cutoff value used to discriminate more than two non-WC components of MS was 0.44 in boys, with a J-value of 0.323 (sensitivity, 0.67; specificity, 64.6%) and an AUC of 0.691, and 0.43 in girls, with a J-value of 0.333 (sensitivity, 66.4%; specificity, 66.9%) and an AUC of 0.684 (Table 2). 

When we compared an optimal WHtR cutoff value of 0.43 for the prediction of two or more non-WC components of MS with the values of 0.5 and 0.6, our values were lower. The sensitivity of our analysis was 69.8%, which was higher than that for other cutoff values; however, the specificity of 62.2% was lower than that for other cutoff values, and the positive predictive value of 19.0% was lower than other cutoff values. Similar results were found in both boys and girls (Table 3).

**Figure 1:**
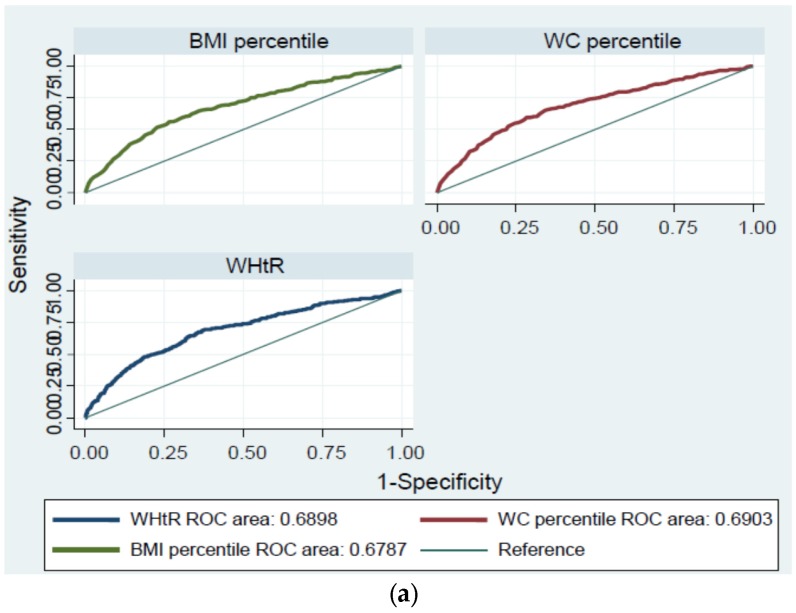

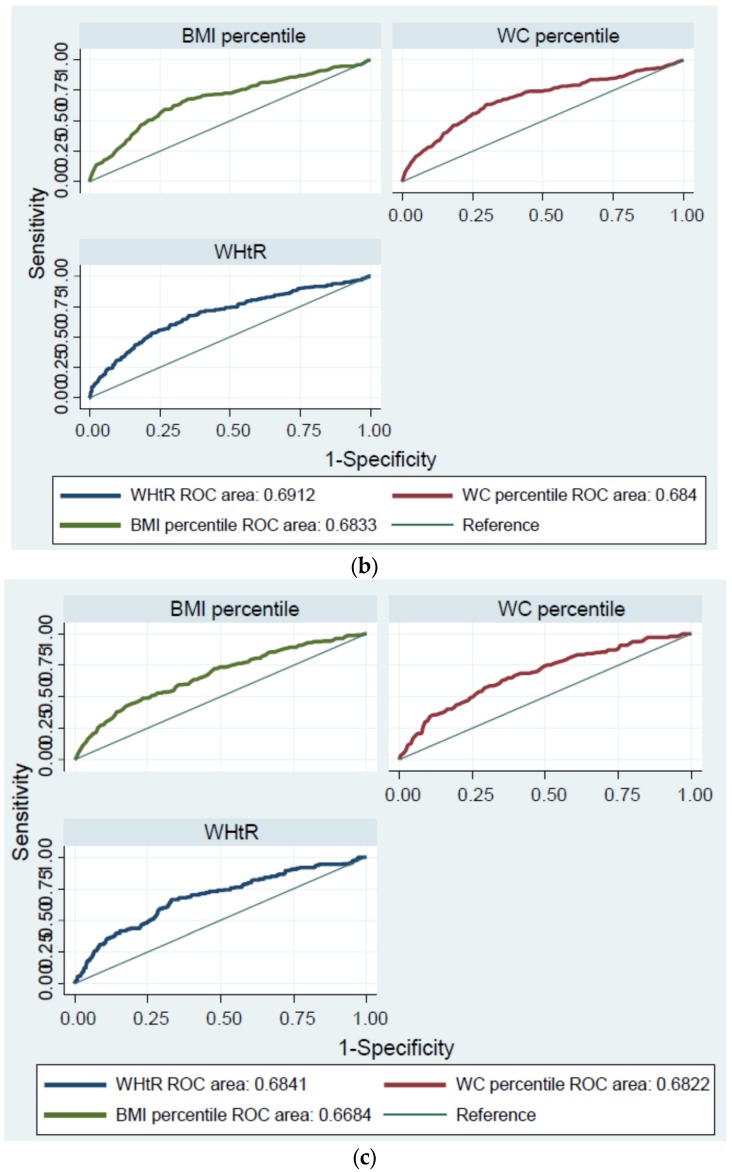
The receiver operating characteristic (ROC) curves for body mass index (BMI) percentile, waist circumference (WC) percentile, and the waist-to-height ratio (WHtR) to predict two or more non-waist circumference components of metabolic syndrome according to sex. (**a**) Total; (**b**) Boys; (**c**) Girls.

Table 4 presents a comparison of the usefulness of WHtR and other obesity indicators (BMI and WC percentile) for the prediction of two or more non-WC components of MS using AUC values. No indicators for predicting high FPG or high BP were statistically significant in either girls or boys. For high triglyceride, WHtR had the highest AUC value of 0.664, and overall the AUCs for WC percentile and WHtR were larger than those for BMI percentile (boys, 0.689; girls, 0.638); however, these differences were not statistically significant. For low HDL-cholesterol, the AUCs for WC percentile were 0.655 for boys and 0.715 for girls, respectively, which were superior to those of other indices, and statistically significant in boys. The AUC for WHtR for two or more non-WC components of MS was 0.690 for total, 0.691 for boys and 0.684 for girls, which were larger than the AUC values for the other two indices, albeit without statistical significance (total, *p* = 0.216; boys, *p* = 0.469; girls, *p* = 0.512) (Table 4, Figure 1). 

Study subjects were divided into two groups based on the WHtR cutoff (0.43) and the MS index and other cardiovascular risk factors were compared (Table S1). In the group with a WHtR above the cutoff value, systolic BP, fasting blood glucose, total cholesterol, triglyceride, and LDL-cholesterol levels were significantly higher and HDL-cholesterol levels lower compared with those in the group with a WHtR below the cutoff value. The rate of central obesity, prevalence of MS risk factors, and rate of MS diagnosis were also significantly higher. Moreover, hemoglobin A1c levels, Glutamic oxaloacetic transaminase (GOT), Glutamic pyruvic transaminase (GPT), and Gamma glutamyl transpeptidase (GGTP) were also significantly higher.

## 4. Discussion

In this study, the optimal WHtR cutoff value for predicting obesity and MS was obtained using data representative of the Korean population. The usefulness of this cutoff value as a screening tool for the prediction of MS was compared with those for BMI and WC, which are conventional obesity indices. The WHtR cutoff value for MS diagnosis was less than the optimal WHtR cutoff value used for general and central obesity screening. WHtR was found to be a very accurate index for obesity screening; however, when comparing AUC values, its accuracy in detecting MS was statistically similar to those of BMI and WC percentiles.

To prevent adult-onset cardiovascular disease, it is important to determine those at high risk for obesity and metabolic abnormalities during childhood and adolescence [24]. BMI and WC are simple and inexpensive screening measurements for predicting obesity and MS, and they are commonly used in adults and children [38]. BMI is associated with body fat content but not always with abdominal obesity [13]. WC may reflect the extent of visceral fat accurately, but it can overestimate or underestimate the risk of cardiovascular disease, as individuals with similar WCs may vary in height [39]. In addition, the above two indicators are inconvenient for screening in children because of various differences in race, age, and sex-dependent aspect [40,41]. WHtR has the advantage of taking into account abdominal obesity as well as height associated with body fat accumulation or distribution [14,42]. Moreover, WHtR was found to be associated with variables relating to body fat, including trunk and body fat percentages, both of which are measured by dual energy X-ray absorptiometry, in both children and adults [43]. Ashwell et al. [44] suggested that WHtR is more sensitive than BMI for early prediction of obesity-related complications, is easy to measure by the general public, and is commonly used for all ages, sexes, and races. WHtR can be assessed in children and adolescents, whose height and WC change as they grow regardless of age [17], and used to evaluate the risk of cardiovascular disease accurately, compared with WC alone, since WHtR also takes into account height [12]. 

There has been controversy as to whether WHtR is independent of race and age [45]. Studies have also been performed to determine if a more accurate cutoff value than 0.5 exists for determining obesity or metabolic disorders in different races and sexes, especially in children and adolescents [23]. In this study, the optimal cutoff WHtR value for predicting MS risk was 0.44 in boys and 0.43 in girls. The optimal cutoff values for diagnosing general obesity and central obesity were 0.47 and 0.5 for boys and 0.47 and 0.48 for girls, respectively, which were higher than the cutoff values for diagnosing MS. Previous studies proposed optimal cutoff values for obesity screening in children and adolescents of 0.51 and 0.49 for Korean boys and girls, respectively [20], 0.47 and 0.45 for Chinese boys and girls, respectively [19], and 0.48 and 0.47 for Australian boys and girls, respectively [21]. Similar to this study, all AUC values were close to 1, which indicates a high predictive ability. The optimal WHtR cutoff value for predicting MS risk in children was 0.41 to 0.44 in Japan [22], 0.52 in the United States [24], and 0.465 and 0.455 for boys and girls in Africa, respectively [23]. In a prospective study of children and adolescents in Australia, the WHtR value used in children to predict cardiometabolic risk factors during adolescence was 0.47 for boys and 0.44 for girls [46]. In previous studies, the optimal cutoff value for MS screening was usually less than 0.5, except in studies conducted in obese children [18,47,48], which is generally consistent with the values presented in the literature [19,23,46,49,50]. There are several reasons why optimal cutoff values differ among studies. First, the diagnostic criteria used to evaluate MS and individual metabolic risk factors may differ [51]. Next, since body fat patterns show ethnic differences, visceral fat distribution associated with cardiometabolic risk may have a variety of effects depending on race [51]. It is also important to note that the prevalence of obesity and MS differ according to subject characteristics, including race, age, and geographic region [23]. Bauer et al. [24], who used an optimal cutoff value of 0.5 or greater, reported an obesity rate of 29.5% and an overall mean WHtR of 0.50, which was higher than the rate in our study. Lastly, different WC measurement methods have been used among studies. Gil et al. [20] reported an optimal WHtR cutoff value corresponding to a BMI ≥the 95th percentile in Korean children. In that study, unlike our method of measurement, the lowest WC value was used, and as a result, the optimal cutoff value was 0.48 for boys and 0.47 for girls. Therefore, for WHtR to be used as an indicator on its own, it is necessary to measure WC uniformly across studies; measuring weight and height in large epidemiological studies and clinical practice should always be encouraged [52,53]. 

Ashewell [54] suggested an optimal WHtR cutoff value of 0.5 for the prediction of obesity and metabolic abnormalities in children and adults and proposed that individuals with values ≥0.5 should be followed up, while immediate action should be taken in individuals with values ≥0.6. The sensitivity and specificity for MS diagnosis using WHtR cutoffs of 0.5 and 0.6 were analyzed. Sensitivity was lower and specificity higher when using these values compared with our optimal cutoffs. When we applied a cutoff value of 0.6, which was proposed in obese children and adolescents, the specificity reached 99.7%, but the sensitivity was only 2.6%. In a prospective study of adolescents in Australia, it was reported that a WHtR cutoff value of ≥0.5 was sufficiently high to identify cardiometabolic risk co-occurrence, but the sensitivity was very low [46]. The lower the WHtR cut-off value, the higher the probability of an incorrect cardiovascular risk factor diagnosis, resulting in unnecessary resources and efforts to manage these risk factors [46]. On the other hand, increasing the cutoff value reduces the probability of misdiagnosing but increases the likelihood that a child with MS is missed [46]. Taking into consideration the critical role that the sensitivity plays in screening for childhood metabolic risk in population health studies involving an extensive number of subjects, it may be necessary to place emphasis on increasing the sensitivity rather than specificity. A cutoff value of 0.5, which is feasible clinically, can be used to predict obesity in Korean children. However, its estimated sensitivity for the detection of metabolic risk is 32% in boys and 20% in girls. For the purpose of screening for early detection and prevention of MS in Korea, it is appropriate to use a general value of 0.43 (boys, 0.44; girls, 0.43) as a cutoff, considering its high sensitivity and negative predictive value. In research of pre-indicators of cardiovascular disease, this study demonstrates perturbed children’s metabolic profile since those with a WHtR above the cutoff value (0.43) are likely to exhibit adverse clinical factors beyond obesity and MS as shown in supplementary Table S1. In such context, screening children with a general cutoff value of 0.43 (boys, 0.44; girls, 0.43) is appropriate for early detection and prevention of MS in Korea, considering its high sensitivity and negative predictive value.

Previous studies have shown that WHtR is either a more appropriate [22,55,56,57,58], similar [59,60], or inferior [27,38] method than is BMI for screening for metabolic abnormalities. In this study, except for low HDL cholesterol values in girls, the AUCs of all indices were less than 0.7, indicating mediocre results. WHtR performed better than did BMI and WC percentiles as a screening tool for the prediction of two or more non-WC components of MS, but there was no statistically significant difference. The best predictors for high triglyceride were WC and WHtR, and the best predictor for low HDL cholesterol was WC in both boys and girls. These results suggest that WC and WHtR, which better reflect abdominal obesity, have a higher AUC than that for BMI, as triglycerides and HDL cholesterol are highly correlated with the amount of intra-abdominal fat in adolescents [61]. Lo et al. [12] conducted a meta-analysis of cross-sectional studies and found that the AUCs for high BP, high triglyceride, and low HDL cholesterol levels were <0.7, but the AUCs for the prediction of MS was ≥0.7 in adolescents. The majority of the outcomes had very high heterogeneity [12]. This suggests that the prevalence of MS is generally low in children and adolescents, and the prevalence of a high BP, high triglyceride or glucose level, low HDL cholesterol level, and MS varies according to the study subjects. However, a meta-analysis of adults showed that WHtR was superior to BMI and WC as an indicator of risk factors for cardiovascular metabolic disease, and the calculated AUC value was ≥0.7 [16]; similar results were obtained in a study investigating Korean adults [62]. In a meta-analysis of cohort studies, WHtR was demonstrated to be superior to BMI in detecting outcomes such as incidence of cardiovascular disease and cardiovascular or all-cause mortality, particularly in Asians [63]. The reason why the efficacy of WHtR is lower in children and adolescents compared with adults may be explained by variations in WHtR according to age [12,64]. As children become adolescents, increases in height are relatively greater than increases in WC, which suggests the potential for misclassification of children with excess abdominal fat as healthy [12]. It is also important to note that the prevalence of MS is lower in children than in adults [23]. Since childhood obesity is related to various metabolic factors, biochemical abnormalities usually do not appear until later in life, and the morbidity rate is lower in children than in adults [23,65]. In addition, it is also possible that high lipid profiles may be related to family history rather than weight in pediatric individuals [66]. Nevertheless, WHtR is an excellent tool to replace the existing obesity index because the AUC values of WHtR for detecting obesity defined by BMI and WC converge to 1. Use of WHtR also resulted in an accurate prediction of risk factors for cardiovascular metabolic disease, to the same extent as BMI and WC. Because WHtR is easy to measure and apply clinically and for the general public to comprehend, it can be used in place of the existing obesity index to identify children and adolescents at risk of cardiometabolic disorders at an early age.

This study is not without limitations. First, it was a cross-sectional study; therefore, it did not provide enough evidence to determine the predictive value of WHtR for metabolic risk. However, it provided diagnostic value for obesity and MS. Future cohort studies using a prospective design are warranted to evaluate the validity of the obtained cutoff values. Next, the cutoff values obtained in this study cannot be applied to other ethnic groups. The prevalence of obesity and MS in children and adolescents were lower than those in other countries, which may have affected the results. Third, “complex sample analysis” could not be performed for ROC curve analysis because of limitations in the statistical program used; however, we performed ROC curve analysis for BMI and WC percentiles by stratifying according to age. Finally, the puberty status of the study population was not used because of the lack of collected date, although sexual maturity is related to obesity or metabolic diseases in youth [67]. 

To our knowledge, this is the first study to examine the utility of WHtR as a screening tool for MS in Korean children and adolescents. The strengths of our study include a comparison of various anthropometric indices and the identification of WHtR as a screening tool for obesity and MS among children and adolescents, using a sample representative of the Korean population. Unlike WHtR, which changes only slightly with age [59], BMI is usually lowest at 5–6 years of age and subsequently increases in proportion to weight and height until adolescence [68]. WC is characterized by a relatively constant increase [68]. Therefore, it is important to perform ROC curve analysis after stratifying by age for BMI and WC; the percentiles were calculated to compensate for errors associated with low percentiles in younger subjects with a low probability of metabolic abnormalities and relatively low WC and BMI values. 

Since early detection of individuals at risk for MS is important for prevention and appropriate interventions [24], future prospective cohort studies should be performed to continue investigating the WHtR cutoff and its usefulness in predicting MS.

## 5. Conclusions

In this study, WHtR was shown to be a simple and effective measurement for screening for childhood obesity and MS in Korean children and adolescents. The optimal WHtR cutoff point for MS was 0.44 in boys and 0.43 in girls, which is slightly less than half of the height (0.5). Considering that today children and adolescents have a long life expectancy, early detection and management of metabolic risk are crucial. Therefore, the optimal WHtR cutoff value presented in this study is of value in the field of public health, as it is easy to use for screening for MS risk in large-scale epidemiological studies and in clinical practice.

## Figures and Tables

**Table 1:** Baseline characteristics of study subjects.

	Total (*n* = 3057)	Boys (*n* = 1625)	Girls (*n* = 1432)	*p*-Value
Age (years)	14.26 ± 0.06	14.26 ± 0.07	14.26 ± 0.09	0.965
Height (cm)	161.69 ± 0.25	165.25 ± 0.38	157.61 ± 0.25	<0.001 **
Weight (kg)	54.80 ± 0.32	58.03 ± 0.49	51.10 ± 0.36	<0.001 **
BMI, (kg/m^2^)	20.71 ± 0.08	20.96 ± 0.12	20.42 ± 0.11	0.001 **
WC (cm)	69.45 ± 0.22	71.33 ± 0.33	67.29 ± 0.29	<0.001 **
WHtR	0.430 ± 0.00	0.432 ± 0.00	0.427 ± 0.00	0.038 *
Systolic BP (mmHg)	107.22 ± 0.25	109.56 ± 0.34	104.54 ± 0.33	<0.001 **
Diastolic BP (mmHg)	66.33 ± 0.23	66.72 ± 0.32	65.88 ± 0.28	0.035 *
FPG (mg/dL)	89.15 ± 0.18	89.49 ± 0.22	88.76 ± 0.22	0.006 **
Total cholesterol (mg/dL)	157.75 ± 0.66	153.03 ± 0.85	163.16 ± 0.87	<0.001 **
Triglyceride (mg/dL)	83.15 ± 1.22	81.67 ± 1.58	84.82 ± 1.64	0.135
HDL cholesterol (mg/dL)	49.97 ± 0.25	48.53 ± 0.29	51.62 ± 0.34	<0.001 **
LDL cholesterol (mg/dL)	91.92 ± 0.55	88.98 ± 0.73	95.28 ± 0.75	<0.001 **
Obesity (BMI percentile ≥95 or BMI ≥25 kg/m^2^) (%)	12.0 (0.7)	14.1 (1.1)	9.7 (1.1)	0.009 **
Overweight (BMI percentile 85–94) (%)	6.0 (0.5)	4.4 (0.5)	7.9 (0.8)	<0.001 **
Normal weight (BMI percentile 5–84) (%)	76.2 (0.9)	75.6 (1.3)	76.9 (1.3)	0.519
Underweight (BMI percentile <5) (%)	5.8 (0.5)	6.1 (0.8)	5.5 (0.7)	0.582
Central obesity (WC ≥the 90th percentile) (%)	8.9 (0.6)	7.6 (0.8)	10.5 (1.0)	0.026 *
High BP (%)	25.6 (1.0)	26.7 (1.4)	24.2 (1.3)	0.166
High FPG (%)	0.5 (0.1)	0.6 (0.2)	0.4 (0.2)	0.604
High triglyceride (%)	19.7 (1.0)	20.3 (1.3)	19.0 (1.3)	0.435
Low HDL cholesterol (%)	14.5 (0.8)	17.9 (1.2)	10.7 (1.1)	<0.001 **
At least one non-WC components of MS ^†^ (%)	45.6 (1.2)	48.4 (1.5)	42.5 (1.6)	0.005 **
Two or more non-WC components of MS ^†^ (%)	12. (0.8)	14.4 (1.2)	10.2 (1.0)	0.007 **
MS (%)	6.2 (0.5)	7.0 (0.8)	5.2 (0.8)	0.102

BMI: body mass index; WC: waist circumference; WHtR: waist circumference-height ratio; BP: blood pressure; FPG: fasting plasma glucose; HDL: high density lipoprotein; LDL: low density lipoprotein; MS: metabolic syndrome; Data expression as estimated mean ± standard error or estimated percent (standard error), as appropriate; * *p* < 0.05, ** *p* < 0.01 (*p*-value were analyzed by chi-square test or *t*-test.); ^†^ The non-WC components of MS were defined according to modified National Cholesterol Education Program, Adult Treatment Panel III (NCEP ATP III) criteria (1) triglycerides ≥110 mg/dL; (2) HDL cholesterol <40 mg/dL; (3) systolic or diastolic BP ≥90th percentile; (4) fasting plasma glucose level ≥110 mg/dL.

**Table 2:** Results of ROC curve analysis to identify optimal WHtR to predict overweight, obesity, central obesity, and two or more non-waist circumference components of metabolic syndrome among children and adolescents.

	AUC	Cutoff Values ^‡^	Sensitivity (%)	Specificity (%)	J Value ^†^	*p*-Value
**Total (*N* = 3057)**						
Overweight (BMI ≥ 85th percentiles)	0.846	0.44	93.1	70.5	0.636	<0.001 **
Obesity (BMI ≥ 95th percentiles or BMI ≥ 25 kg/m^2^)	0.957	0.47	92.6	87.1	0.767	<0.001 **
WC (≥75th percentiles)	0.973	0.45	93.9	90.2	0.841	<0.001 **
WC (≥90th percentiles)	0.978	0.48	96.4	90.6	0.870	<0.001 **
High BP ^a^	0.513	0.49	18.5	87.6	0.061	0.298
High FPG ^b^	0.623	0.43	68.8	61.0	0.297	0.167
High triglyceride ^c^	0.664	0.43	62.2	64.8	0.270	<0.001 **
Low HDL cholesterol ^d^	0.658	0.44	56.0	71.4	0.273	<0.001 **
Two or more non-WC components of MS	0.690	0.43	69.8	62.2	0.320	<0.001 **
**Boys (*n* = 1625)**						
Overweight (BMI ≥ 85th percentiles)	0.861	0.45	95.8	70.2	0.660	<0.001 **
Obesity (BMI ≥ 95th percentiles or BMI ≥ 25 kg/m^2^)	0.950	0.47	91.1	85.9	0.770	<0.001 **
WC (≥75th percentiles)	0.978	0.46	96.9	89.9	0.868	<0.001 **
WC (≥90th percentiles)	0.990	0.50	97.5	94.4	0.919	<0.001 **
High BP ^a^	0.523	0.50	17.7	89.4	0.071	0.191
High FPG ^b^	0.621	0.43	70.0	58.4	0.284	0.206
High triglyceride ^c^	0.689	0.44	63.3	66.2	0.296	<0.001 **
Low HDL cholesterol ^d^	0.631	0.44	56.3	67.8	0.240	<0.001 **
Two or more non-WC components of MS	0.691	0.44	67.7	64.6	0.323	<0.001 **
**Girls (*n* = 1432)**						
Overweight (BMI ≥ 85th percentiles)	0.856	0.44	90.2	75.5	0.657	<0.001 **
Obesity (BMI ≥ 95th percentiles or BMI ≥ 25 kg/m^2^	0.965	0.47	92.7	90.2	0.829	<0.001 **
WC (≥75th percentiles)	0.980	0.44	94.7	89.9	0.846	<0.001 **
WC (≥90th percentiles)	0.985	0.48	94.6	94.6	0.892	<0.001 **
High BP ^a^	0.501	0.49	14.7	90.9	0.056	0.9474
High FPG ^b^	0.611	0.49	50.0	92.4	0.424	0.5554
High triglyceride ^c^	0.638	0.43	58.0	67.8	0.258	<0.001 **
Low HDL cholesterol ^d^	0.699	0.44	60.8	71.2	0.320	<0.001 **
Two or more non-WC components of MS	0.684	0.43	66.4	66.9	0.333	<0.001 **

ROC: receiver operating characteristic; AUC: area under curve; BMI: body mass index; WC: waist circumference; WHtR: Waist circumference-height ratio; BP: blood pressure; FPG: fasting plasma glucose; HDL: high density lipoprotein; MS: metabolic syndrome; * *p* < 0.05, ** *p* < 0.01 (Null hypothesis: area = 0.5); ^†^ J = sensitivity + specificity − 1; ^‡^ The optimal cut off point was obtained from Youden index as [maximum (J = sensitivity + specificity − 1)]; ^a^ High BP was diagnosed if systolic or diastolic blood pressure ≥90th percentile; ^b^ High FPG was diagnosed if FPG was ≥110 mg/dL; ^c^ High triglyceride was diagnosed if triglycerides was ≥110 mg/dL; ^d^ Low HDL cholesterol was diagnosed if HDL cholesterol was <40 mg/dL.

**Table 3:** Sensitivity and specificity of WHtR to detect two or more non-waist circumference components of metabolic syndrome.

		Cutoff Values ^†^	Sensitivity (%)	Specificity (%)	PPV (%)	NPV (%)	J Value *
**Total (*N* = 3057)**							
WHtR	Optimal	0.43	69.8	62.2	19.0	94.2	0.320
		0.5	27.8	91.6	29.1	90.9	0.189
		0.6	2.6	99.7	52.9	89.0	0.023
**Boys (*n* = 1625)**							
WHtR	Optimal	0.44	67.7	0.646	21.5	93.3	0.323
		0.5	32.4	0.889	29.5	90.2	0.212
		0.6	4.4	0.996	60.0	87.9	0.040
**Girls (*n* = 1432)**							
WHtR	Optimal	0.43	66.4	66.9	17.9	94.8	0.333
		0.5	20.0	94.4	28.0	91.6	0.144
		0.6	0.7	99.9	33.3	90.3	0.006

WHtR: Waist circumference-height ratio; PPV: positive predictive value; NPV: negative predictive value; * J = sensitivity + specificity – 1; ^†^ The optimal cut off point was obtained from Youden index as [maximum (J = sensitivity + specificity − 1)].

**Table 4:** Area under the ROC curve of obesity indices to predict the presence of non-waist circumference components of metabolic syndrome according to sex.

	BMI Percentile	WC Percentile	WHtR
	AUC (95% CI)	AUC (95% CI)	AUC (95% CI)
**Total (*N* = 3057)**			
High BP ^#^	0.515 (0.491–0.540)	0.502 (0.477–0.527)	0.513 (0.488–0.538)
High FPG ^†^	0.668 (0.504–0.832) *	0.653 (0.480–0.825)	0.623 (0.449–0.796)
High triglyceride ^‡^	0.654 (0.629–0.679) **	0.659 (0.634–0.684) **	0.664 (0.639–0.689) **
Low HDL cholesterol ^§^	0.668 (0.639–0.698) **	0.687 (0.658–0.715) **	0.658 (0.628–0.689) **
At least one non-WC components of MS	0.600 (0.580–0.620) **	0.592 (0.572–0.612) **	0.592 (0.572–0.613) **
Two or more non-WC components of MS	0.679 (0.647–0.710) **	0.690 (0.659–0.721) **	0.690 (0.658–0.721) **
**Boys (*n* = 1625)**			
High BP ^#^	0.528 (0.494–0.562)	0.515 (0.481–0.549)	0.523 (0.489–0.557)
High FPG ^†^	0.670 (0.482–0.857)	0.654 (0.457–0.851)	0.621 (0.434–0.808)
High triglyceride ^‡^	0.678 (0.644–0.712) **	0.689 (0.655–0.723) **	0.689 (0.656–0.723) **
Low HDL cholesterol ^§^	0.649 (0.612–0.686) **	0.655 (0.617–0.693) **	0.631 (0.592–0.669) **
At least one non-WC components of MS	0.612 (0.584–0.639) **	0.606 (0.578–0.634) **	0.599 (0.571–0.627) **
Two or more non-WC components of MS	0.683 (0.642–0.725) **	0.684 (0.641–0.727) **	0.691 (0.650–0.732) **
**Girls (*n* = 1432)**			
High BP ^#^	0.501 (0.466–0.536)	0.522 (0.486–0.557)	0.501 (0.465–0.537)
High FPG ^†^	0.646 (0.316–0.976)	0.612 (0.242–0.981)	0.611 (0.241–0.981)
High triglyceride ^‡^	0.629 (0.591–0.660) **	0.638 (0.601–0.674) **	0.638 (0.600–0.675) **
Low HDL cholesterol ^§^	0.690 (0.642–0.738) **	0.715 (0.670–0.761) **	0.699 (0.652–0.747) **
At least one non-WC components of MS	0.585 (0.555–0.615) **	0.573 (0.543–0.603) **	0.583 (0.553–0.613) **
Two or more non-WC components of MS	0.668 (0.620–0.717) **	0.682 (0.635–0.730) **	0.684 (0.636–0.733) **

ROC: receiver operating characteristic; AUC: area under curve; CI: confidence interval; BMI: body mass index; WC: waist circumference; WHtR: waist circumference-height ratio; BP: blood pressure; FPG: fasting plasma glucose; HDL: high density lipoprotein; MS: metabolic syndrome; * *p* < 0.05; ** *p* < 0.01 (Null hypothesis: area = 0.5); ^#^ High BP was diagnosed if systolic or diastolic blood pressure ≥90th percentile; ^†^ High FPG was diagnosed if FPG ≥110 mg/dL; ^‡^ High triglyceride was diagnosed if triglycerides was ≥110 mg/dL; ^§^ Low HDL cholesterol was diagnosed if HDL cholesterol was <40 mg/dL.

## Supplementary Materials

The following are available online at http://www.mdpi.com/2072-6643/9/3/256/s1, Table S1: The comparison of various clinical factors according to the waist circumference-to-height ratio (WHtR) cut-off value.
