# Supplementary material for: Usefulness of the Waist Circumference-to-Height Ratio in Screening for Obesity and Metabolic Syndrome among Korean Children and Adolescents: Korea National Health and Nutrition Examination Survey, 2010–2014
Source: Nutrients. 2017 Mar 10;9(3):256. doi: 10.3390/nu9030256 (PMC5372919; doi:10.3390/nu9030256)
Supplement: Supplementary file 1 [file nutrients-09-00256-s001.docx]

Supplementary Materials: Usefulness of the waist circumference-to-height ratio in screening for obesity and metabolic syndrome among Korean children and adolescents: Korea National Health and Nutrition Examination Survey, 2010–2014

**Dong-Hyun Choi, Yang-Im Hur, Jae-Heon Kang, Kyoungwoo Kim, Young Gyu Cho, Soo-Min Hong, Eun Byul Cho**

**Table S1.** The comparison of various clinical factors according to the waist circumference-to-height ratio (WHtR) cut-off value

|  | **≤ 0.43**  **(n=1791)** | **> 0.43**  **(n=1266)** | ***p*-value** |
| --- | --- | --- | --- |
| Age, years | 14.26±0.07 | 14.26±0.10 | 0.986 |
| Height, cm | 161.92±0.33 | 161.37±0.40 | 0.293 |
| Weight, kg | 49.37±0.28 | 62.36±0.55 | <.001** |
| BMI, kg/m^2^ | 18.62±0.06 | 23.61±0.12 | <.001** |
| WC, cm | 63.81±0.16 | 77.28±0.31 | <.001** |
| Systolic BP, mmHg | 105.47±0.30 | 109.66±0.36 | <.001** |
| Diastolic BP, mmHg | 66.11±0.27 | 66.63±0.35 | 0.213 |
| FPG, mg/dl | 88.55±0.20 | 89.98±0.27 | <.001** |
| Total cholesterol, mg/dl | 154.32±0.75 | 162.53±1.06 | <.001** |
| Triglyceride, mg/dl | 72.90±1.19 | 97.40±2.12 | <.001** |
| HDL cholesterol, mg/dl | 51.63±0.31 | 47.66±0.38 | <.001** |
| LDL cholesterol, mg/dl | 88.21±0.63 | 97.071±0.89 | <.001** |
| Hemoglobin A1c (%) | 5.44±0.01 | 5.49±0.01 | <.001** |
| AST, U/l | 17.98±0.14 | 19.99±0.32 | <.001** |
| Elevated AST > 40 U/l (%) | 0.53(0.26) | 3.20(0.58) | <.001** |
| ALT, U/l | 11.54±0.13 | 19.73±0.68 | <.001** |
| Elevated ALT > 40 U/l | 0.34(0.15) | 6.85(0.86) | <.001** |
| GGTP, U/l | 13.89±0.18 | 17.84±0.48 | <.001** |
| Elevated GGTP > 50 U/l | 0.14(0.10) | 0.98(0.36) | 0.004** |
| Central obesity (%)(75) | 0.11(0.08) | 49.43(1.79) | <.001** |
| Central obesity (%)(90) | . | 21.31(1.39) | . |
| High BP (%) | 23.80(1.33) | 28.0(1.55) | 0.033 * |
| High FPG (%) | 0.33(0.18) | 0.75(0.25) | 0.180 |
| High triglyceride (%) | 12.52(1.03) | 29.69(1.69) | <.001** |
| Low HDL cholesterol (%) | 9.34(0.90) | 21.77(1.47) | <.001** |
| At least one nonadipose MS (%) | 38.91(1.43) | 55.03(1.84) | <.001** |
| Two or more nonadipose MS (%) | 6.36(0.77) | 20.89(1.39) | <.001** |
| MS (%) | 0.72(0.30) | 13.77(1.18) | <.001** |

BMI: body mass index; WC: waist circumference; WHtR: Waist circumference-height ratio; BP: blood pressure; FPG: fasting plasma glucose; HDL: high density lipoprotein; LDL: low density lipoprotein; AST: aspartate transaminase; ALT: alanine transaminase; GGTP: gamma glutamyl transpeptidase; MS: metabolic syndrome. Data expression as estimated mean ± standard error or estimated percent (standard error), as appropriate. * *p* < 0.05; ** *p* < 0.01 (*p*-value were analyzed by chi-square test or t-test). The diagnostic value of WHtR with suggested cut-offs in the evaluation of metabolic syndrome in children and adolescents in a cross-sectional study.
